# A Comparative Study Between Vacuum Dressing and Normal Saline Dressing for Chronic Non-Healing Ulcers

**DOI:** 10.7759/cureus.23870

**Published:** 2022-04-06

**Authors:** Pragadheeswaran M, Sankar lingam P, Yuvaraj Balan, Anand K Pyati

**Affiliations:** 1 General Surgery, Mundiyampakkam Medical College and Hospital, Villupuram, Tamil Nadu, IND; 2 General Surgery, Chengalpattu Medical College and Hospital, Chengalpattu, Tamil Nadu, IND; 3 Biochemistry, All India Institute of Medical Sciences, Bibinagar, Hyderabad, IND

**Keywords:** normal saline dressing, diabetic ulcer, negative pressure therapy, topical negative pressure therapy, sub-atmospheric pressure therapy, chronic wound, negative pressure dressing, vacuum dressing, non-healing ulcer, healing ulcer

## Abstract

Introduction

Isn’t it a boon that all living organisms possess the ability to heal their injuries? The wound healing is faster when the normal physiology of the wound healing is maintained. Our understanding of wound healing has undergone dramatic changes in the recent past. Almost all materials and methods available on earth have been used and tested to facilitate the process of wound healing. The mental agony and the disability suffered by patients with chronic ulcers have led to the reappraisal of the basic components of the wound healing process and how they are influenced by biological, mechanical, and physical forces.

The Department of General Surgery in our Government Chengalpattu Medical College and Hospital, Chengalpattu, Tamil Nadu, India, admits and treats a large volume of patients with wounds and ulcers. Here many materials are being used regularly for dressing to make wound healing faster. Vacuum dressings were also done on many patients, and promising results were observed. This kindled our interest in conducting this prospective study and comparing wound healing with vacuum dressing versus normal saline dressing.

Materials and methods

A total of 74 patients were included in the study, out of which 37 patients were randomly included in the experimental group and vacuum dressing was done, while the other 37 included in the control group were treated with dressing done with normal saline moistened gauze and bandage roll. Rates at which the wound healed were compared.

Results

We were able to observe a statistically significant difference in the rate of appearance of granulation tissue between the two and increased clearance of bacteria and toxins. The study group promised better progress as compared to the control group in various aspects.

Conclusion

Vacuum dressing brings an obvious improvement in the healing of non-healing ulcers and decreases the overall duration of stay in the hospital.

## Introduction

“Chronic wounds” are commonly defined as “wounds that have not proceeded through an orderly and timely reparation to produce anatomic and functional integrity after 3 months. Leaper and Durani defined it as a wound that lacks a 20-40% reduction in size after two to four weeks of optimal treatment or when there is no complete healing after six weeks [1]. These ulcers remain in the inflammatory stage for a very long period and never heal [2]. These ulcers have a great impact on the social as well as the economic status of the patient and the family [3].

In Canada, it costs approximately more than $100 million annually for the community care of treating leg ulcers of all etiologies [4]. In the USA, in 2009, more than 6.5 million patients suffered from chronic wounds, which cost US$25 billion to the healthcare system [5]. UK's National Health Service treated 2.2 million patients with wounds, and £5.3 billion was spent as per the study of 2012-2013 [6]. In 2014, The Australian Wound Management Association (AWMA) estimated the number of patients with chronic ulcers to be 420,000, and the estimated annual cost to treat them was AUS$3.5 billion [7]. In the Scandinavian countries, associated wound care costs account for 2-4% of the total healthcare expenditure [8,6]. A cost-modeling study conducted in Denmark suggests that chronic wounds cost DKK56 million in 2009 [9]. In developing countries like India, approximately 3-4% of all people with diabetes have a foot problem and use 12-15% of the healthcare resources [10].

Non-healing ulcers may be due to complications of an underlying disease, such as diabetes mellitus or following surgery, trauma, or burns, or may also be due to constant pressure. These are more commonly found in the elderly with chronic diseases, with the most common being diabetes and the organ frequently affected being the foot [4]. According to a study, as high as 25% of patients with diabetes mellitus may develop foot ulcers in their lifetime [11].

Various materials and techniques have been used in fastening the healing process [12,13]. In this study, the application of vacuum (Negative pressure) to the wound was also studied. The idea of applying vacuum to a wound in a closed environment was developed in the 1980s and 1990s by groups including Chariker and Jeter describing a gauze-based system [14]. A German group led by Fleischmann et al. reported the management of patients with open fractures using a foam dressing [15]. Morykwas and Argenta are the ones who published first a modality with the application of a controlled vacuum using an open-cell foam dressing for the treatment of acute and chronic wounds in 1997 [16-18].

Subatmospheric pressure therapy, topical negative-pressure therapy, and vacuum sealing/vacuum pack therapy are other names for vacuum dressing. The mechanism of action of vacuum therapy is an interplay of factors that involve clearance of bacteria and toxins, maintaining appropriate moisture, enhancing vascularity and blood flow, and facilitating wound granulation, thus promoting wound healing. There is also evidence for vacuum dressing in increasing dermal perfusion, decreasing the interstitial fluid accumulation (edema reduction), and playing a significant role in tissue salvage [19].

Some randomized trials have been conducted in the recent past and proved the efficacy of the vacuum dressing on wounds [20-23]. A randomized trial by Apelqvist et al. showed that there was a utilization of fewer resources and that a greater proportion of patients showed wound healing at a lower overall cost of care as compared to routine dressing [24]. The data on the role of a vacuum dressing for non-healing ulcers are limited [25]. Hence, we conducted a study comparing the efficacy between vacuum dressing and normal saline-moistened gauze dressing for chronic non-healing ulcers.

## Materials and methods

Ethical committee approval

Approval was requested from the Institutional Ethical Committee, Chengalpattu Medical College, Chengalpattu, Tamil Nadu, India (ECR/774/INST/TN/2015).

The request for approval from the Institutional Ethical Committee (IEC) was considered at the IEC meeting held on September 20, 2017, at the Medical Education Unit, Government Medical College, Chengalpattu. The members of the committee, the secretary, and the chairman approved the proposed project.

Following the approval, we conducted this prospective study on patients with chronic non-healing ulcers in the Department of General Surgery, Chengalpattu Medical College and Hospital, between October 2017 and October 2018 for a period of one year.

Aims and objectives of the study

Our primary objective is to compare the observations between vacuum dressing and normal saline dressing in patients with chronic non-healing ulcers in terms of the time taken for complete ulcer healing/ulcer bed preparation (healthy granulation); their capabilities to prevent/control infection; the differences in the morbidity suffered by the patient assessed in terms of pain perceived (subjectively), discharge, smell, and the ability to ambulate; and overall duration of hospitalization.

Inclusion criteria

Patients with chronic ulcers (ulcers for more than six weeks’ duration) due to diabetes, trauma, pressure sores, and necrotizing fasciitis who were willing to give informed consent and above 14 years of age were included in the study.

Exclusion criteria

Patients with non-healing ulcers but admitted to the intensive care unit (ICU) with other complications such as septic shock, diabetic ketoacidosis, or kidney injuries, and patients whose vitals were unstable were considered critically ill and were excluded. Patients with any evidence of underlying bone osteomyelitis and malignancy were also not included in the study.

Commencement of the study

Initially, we prepared a protocol and a pro forma chart. As per the protocol, chronic ulcer patients after admission to the ward were carefully examined and their general conditions were stabilized. Secondly, the following aspects were assessed clinically: the size and location of the ulcer, infection present or absent, debridement needed or not, and treatment modality (vacuum dressing or normal saline dressing).

Based on the inclusion criteria and exclusion criteria, eligible patients were carefully selected. Then the patients were clearly explained about the treatment modality, and consent was obtained. In total, 74 patients were included in the study, out of which 37 patients were randomly included in the experimental group and vacuum dressing was done, while the other 37 were included in the control group and were treated with normal saline dressing.

Initial management common to both groups

The patients after admission were subjected to detailed clinical examinations with baseline investigations including antibodies against HIV1 and HIV2 tests (human immunodeficiency virus), detection of hepatitis B surface antigen (HBsAg), and hepatitis C antibody test (AntiHCV). X-rays of local parts were taken to rule out osteomyelitis. They were started on empirical antibiotics. They were also posted for surgical procedures at the emergency operating theater whenever required, and wound debridement or cleaning of the wound was done and then a dressing was done using gauze pad and roller bandage. On the next day, they were transferred to the general ward. A protein-rich diet was started as soon as possible to improve the general condition. The dressing was removed, and a wound swab for bacterial growth and culture sensitivity was sent. The patients were asked to wash the wound with soap foam. They were also motivated to bathe daily if possible. The patients were also encouraged to walk as soon as possible. A wound swab was sent every fourth day, and according to the culture sensitivity report, the antibiotics were changed.

Treating the control group

The wounds of the patients in the control group were cleaned with normal saline, hydrogen peroxide, and povidone-iodine solution, and then dressing was done using normal saline moistened gauze and roller bandage. The dressing was done once a day or more frequently if needed.

Treating the experimental group

The wounds of the patients in the experimental group were also thoroughly washed with normal saline and hydrogen peroxide. One end of a perforated drain tube was taken and kept over the wound bed and another end was brought out. Hydrocolloid foam was cut and made to fit the dimensions of the wound, and the wound along with the drain tube was covered. The wound containing the foam and the surrounding normal skin for about 10 cm circumferentially was covered with a semi-permeable, transparent membrane. The membrane was tightened to the skin with adhesive tape. A negative pressure ranging from 80 to 120 mmHg was applied to the other end of the drain tube by connecting it to a suction device. The pressure was almost continuously applied unless the patient wanted to ambulate or wanted a break.

Common for both groups

For diabetic patients, glycemic control was achieved with diet modification, Injection insulin was added, and its dose was titrated once in two to three days. Patients with anemia were transfused with blood and blood products (injection iron sucrose or oral ferrous sulfate tablets and vitamin supplements whenever necessary). Improvement of the ulcer will be recorded once in two days. Time taken for wound healing, the period for which antibiotics were given, and the follow-up period were noted. After the wound had completely epithelialized, they were discharged. For larger ulcers, when their beds were prepared adequately, closure with split skin graft or flap cover was done.

## Results

A total of 74 patients were studied, of whom 37 patients were randomly allocated to the experimental group and were treated with vacuum dressing, while the other 37 were included in the control group and treated with normal saline dressing. The observations are shown in Table 1.

**Table 1:** Comparison of duration for wound healing and overall hospitalization for both groups

Parameter	Vacuum dressing (mean ± SD)	Normal saline dressing (mean ± SD)	p-Value	Conclusion
Duration of wound healing	15.46±3.42	23.95±4.46	0.008	There is a highly significant decrease in the number of days taken for wound healing.
Overall hospitalization	24.75±3.34	34.81±4.09	<0.001	There is a highly significant decrease in the number of days of hospitalization.

Many of the patients in the experimental group who underwent vacuum dressing showed accelerated wound healing. The number of days (mean ± SD) in which the ulcer healed completely/ulcer bed preparation was adequate for the patients in the experimental group, 15.46 ± 3.42 days, whereas, in the control group, similar progress was attained in 23.95 ± 4.46 days, (p = 0.008), as shown in Table 1.

The overall number of days (mean ± SD) the patients in the experimental group stayed in the hospital was 24.75 ± 3.34 days, whereas the patient in the control group stayed for 34.81 ± 4.09 days, (p < 0.001), as shown in Table 1.

In both groups, it was an infection that inhibited wound healing in most of the patients. In the experimental group, 92% had no infection, whereas in the control group, 54% of the cases were free from infection (92% vs 54%, p = 0.0002), as shown in Table 2. This shows a highly significant control of the infection in the experimental group. In both groups, the most common organism found was *Staphylococcus aureus*, and most of the cultures were sensitive to Injection cefotaxime.

**Table 2:** Comparison of other parameters between both groups.

Parameter	Vacuum dressing	Normal saline dressing	p-Value	Chi-square	Conclusion
Infection	3	17	0.0002	13.43	There is a highly significant infection control in the experimental group.
Pain	20	21	0.81	0.06	There is no significant difference.
Discharge	3	18	0.0001	14.96	There is a highly significant decrease in discharge in the control group.
Smell	2	9	0.0222	5.23	There is a significant decrease in the smell in the experimental group.
Ability to ambulate	20	24	0.3435	0.9	There is no significant difference.

Overall, 54% of the patients in the experimental group and 57% of the patients in the control group experienced pain. Thus, concerning pain, there was no significant difference between both groups (54% vs 57%, p = 0.081), as shown in Table 2.

Around 8% of cases from the experimental group had a discharge, while 49% in the control group suffered from increased discharge (8% vs 49%, p = 0.0001), as shown in Table 2. This shows a highly significant decrease in the discharge in the experimental group.

Around 54% cases in the experimental group could be mobilized early and took care of their daily needs by themselves compared to 65% cases in the control group (54% vs 65%, p = 0.3435). In this regard, there was no significance between both groups.

## Discussion

Non-healing wound is one of the troublesome health issues that affect patients significantly. The healing process may take a longer time in some individuals. Till the wound is healed completely, it is mandatory to protect the wound from dirt and germs. Conventionally, this is prepared by simple wash and dressing of the wound. The dressings also absorb the exudates apart from preventing infection. Over time, many substances were applied to the wound with the intention to make it heal faster. In the 1960s, the concept of moist wound dressing created a revolution in wound care.

Similarly, in the early 1990s, the application of vacuum over the wound was also studied, which showed promising results [15]. Many studies have been conducted comparing vacuum dressing and moist dressing and proved vacuum dressing to be more effective in chronic non-healing ulcers [23]. This is also very clearly evident in our study that the patients in the experimental group had wound healing/ulcer bed preparation faster than the patients in the control group with normal saline dressing. In the experimental group, there was an obvious difference in the rate of appearance of granulation and in the overall hospital stay when compared to the control group. This proves the inherent property of the vacuum dressing. The ulcer when treated with vacuum exerts a mechanical force that drives the granulation spurt. Such mechanical force is not available in normal saline dressing. Fabian et al. [26] in rabbit models and Joseph et al. [27] in humans proved the increase in granulation tissue formation. 

Morykwas et al. with the use of a needle probe laser Doppler flowmetry proved that negative pressure of around 125 mm Hg resulted in a fourfold increase in blood flow using an excisional wound model in pigs [17]. A similar increase in blood flow was also demonstrated in human burns. Further increase in the pressure of more than 200 mm Hg was shown to decrease blood flow [19]. The vacuum system also provides a wonderful mechanism for draining the discharge in the ulcer, thus controlling the infection. Avery et al. conducted microbiological studies with bacterial cultures grown from the punch biopsy specimen from the wound. They could demonstrate a significant decrease in bacterial counts in the punch biopsy specimen of the wounds treated with a vacuum after four days [28]. We were also able to observe a similar decrease in bacterial colonization in the ulcers of the patients in the experimental group in comparison with the control group; 46% of the patients in the control group had wound infection, whereas only 8% of the patients in the experimental group had wound infection. The most important inhibitory factor that delayed the healing process of ulcers in the patient in the control group was wound infection [23].

The inconveniences suffered by the patients in the control group are worth mentioning. These patients had wound infections, more discharge, and smell from the ulcer. The patients in the control group could not ambulate early and thus were dependent on their relatives for carrying out their daily personal activities. The patients in the experimental group performed well in this regard. These patients did not have a need for changing the wound dressing more than once a day as they experienced lesser soakage and also lesser pain. Therefore, they could be mobilized early and were able to take care of themselves. Considering the cost, although vacuum dressing for the experimental group was a bit costlier for the initial setup, this is less when compared to the overall expense spent by the control group. The expense spent on the dressing materials, the manpower for cleaning and dressing, and the income lost both for the patient and their relatives when they had to accompany the patient altogether were costlier than the expense spent on the vacuum dressing. 

Limitations of the study

This prospective randomized control study had its limitations. Blinding was not possible in this study because the difference in treatment is very well obvious. Therefore, subject variation bias is inevitable. Though a sample size of 70 patients is sufficient for statistical analysis, a randomized controlled comparative study conducted among a much larger population may strongly reinforce our findings.

## Conclusions

Vacuum dressing when compared to normal saline dressing accelerates the healing process and decreases the time taken for the complete healing/wound bed preparation in patients with chronic non-healing ulcers. Patient compliance is also better, and the overall hospital stay can be reduced with vacuum dressing. It prevents the incidence of Infection and decreases the progression of infection and other related morbidities. Thus, vacuum dressing could be a better choice for chronic non-healing ulcers.

## References

[1] Kyaw BM, Järbrink K, Martinengo L, Car J, Harding K, Schmidtchen A (2018). Need for improved definition of ”chronic wounds” in clinical studies. Acta Derm Venereol.

[2] Iqbal A, Jan A, Wajid M (2017). Management of chronic non-healing wounds by hirudotherapy. World J Plast Surg.

[3] Augustin M, Brocatti LK, Rustenbach SJ (2014). Cost-of-illness of leg ulcers in the community. Int Wound J.

[4] Medical Advisory Secretariat (2006). Negative pressure wound therapy: an evidence-based analysis. Ont Health Technol Assess Ser.

[5] Sen CK, Gordillo GM, Roy S (2009). Human skin wounds: a major and snowballing threat to public health and the economy. Wound Repair Regen.

[6] Guest JF, Vowden K, Vowden P (2017). The health economic burden that acute and chronic wounds impose on an average clinical commissioning group/health board in the UK. J Wound Care.

[7] Norman RE, Gibb M, Dyer A (2016). Improved wound management at lower cost: a sensible goal for Australia. Int Wound J.

[8] Gottrup F, Holstein P, Jørgensen B, Lohmann M, Karlsmar T (2001). A new concept of a multidisciplinary wound healing center and a national expert function of wound healing. Arch Surg.

[9] Hjort A, Gottrup F (2010). Cost of wound treatment to increase significantly in Denmark over the next decade. J Wound Care.

[10] Gupta S, Sagar S, Maheshwar G, Tripathi S (2021). Chronic wounds: magnitude, socioeconomic burden and consequences. Wounds Asia.

[11] Singh N, Armstrong DG, Lipsky BA (2005). Preventing foot ulcers in patients with diabetes. JAMA.

[12] Vermeulen H, Ubbink DT, Goossens A (2005). Systematic review of dressings and topical agents for surgical wounds healing by secondary intention. Br J Surg.

[13] Singh A, Halder S, Menon GR, Chumber S, Misra MC, Sharma LK, Srivastava A (2004). Meta-analysis of randomized controlled trials on hydrocolloid occlusive dressing versus conventional gauze dressing in the healing of chronic wounds. Asian J Surg.

[14] Chariker ME, Jeter KF, Tintle TE, Bottsford Bottsford, JE JE (1989). Effective management of incisional and cutaneous fistulae with closed suction wound drainage.. Contemp Surg.

[15] Fleischmann W, Strecker W, Bombelli M, Kinzl L (1993). [Vacuum sealing as treatment of soft tissue damage in open fractures]. Unfallchirurg.

[16] Morykwas MJ, Argenta LC (1997). Nonsurgical modalities to enhance healing and care of soft tissue wounds. J South Orthop Assoc.

[17] Morykwas MJ, Argenta LC, Shelton-Brown EI, McGuirt W (1997). Vacuum-assisted closure: a new method for wound control and treatment: animal studies and basic foundation. Ann Plast Surg.

[18] Argenta LC, Morykwas MJ (1997). Vacuum-assisted closure: a new method for wound control and treatment: clinical experience. Ann Plast Surg.

[19] Banwell PE, Téot L (2003). Topical negative pressure (TNP): the evolution of a novel wound therapy. J Wound Care.

[20] Armstrong DG, Lavery LA (2005). Negative pressure wound therapy after partial diabetic foot amputation: a multicentre, randomised controlled trial. Lancet.

[21] Eginton MT, Brown KR, Seabrook GR, Towne JB, Cambria RA (2003). A prospective randomized evaluation of negative-pressure wound dressings for diabetic foot wounds. Ann Vasc Surg.

[22] Blume PA, Walters J, Payne W, Ayala J, Lantis J (2008). Comparison of negative pressure wound therapy using vacuum-assisted closure with advanced moist wound therapy in the treatment of diabetic foot ulcers: a multicenter randomized controlled trial. Diabetes Care.

[23] Kasaraneni P, Rao Y, Satyanavamani G, Poornima D (2016). Comparison of vacuum assisted closure vs conventional moist dressing in the management of chronic wounds. IOSR J Dent Med Sci.

[24] Apelqvist J, Armstrong DG, Lavery LA, Boulton AJ (2008). Resource utilization and economic costs of care based on a randomized trial of vacuum-assisted closure therapy in the treatment of diabetic foot wounds. Am J Surg.

[25] Lone AM, Zaroo MI, Laway BA, Pala NA, Bashir SA, Rasool A (2014). Vacuum-assisted closure versus conventional dressings in the management of diabetic foot ulcers: a prospective case-control study. Diabet Foot Ankle.

[26] Fabian TS, Kaufman HJ, Lett ED (2000). The evaluation of subatmospheric pressure and hyperbaric oxygen in ischemic full-thickness wound healing. Am Surg.

[27] Joseph E, Hamori CA, Bergman S, Roaf E, Swann NF, Anastasi GW (2000). A prospective, randomized trial of vacuum-assisted closure versus standard therapy of chronic nonhealing wounds. Wounds.

[28] Avery C, Pereira J, Moody A, Gargiulo M, Whitworth I (2000). Negative pressure wound dressing of the radial forearm donor site. Int J Oral Maxillofac Surg.

